# The role of CD56^bright^ NK cells in neurodegenerative disorders

**DOI:** 10.1186/s12974-024-03040-8

**Published:** 2024-02-13

**Authors:** Carla Rodriguez-Mogeda, Chaja M. J. van Ansenwoude, Lennart van der Molen, Eva M. M. Strijbis, Reina E. Mebius, Helga E. de Vries

**Affiliations:** 1 Department of Molecular Cell Biology and Immunology, Amsterdam UMC Location Vrije Universiteit Amsterdam, Amsterdam, The Netherlands; 2 Amsterdam Neuroscience, Amsterdam, The Netherlands; 3 MS Center Amsterdam, Amsterdam UMC Location Vrije Universiteit, Amsterdam, The Netherlands; 4 IQ Health Science Department, Radboud University Medical Center, Nijmegen, The Netherlands; 5 Department of Neurology, Amsterdam UMC Location Vrije Universiteit, Amsterdam, The Netherlands; 6 Amsterdam Infection and Immunity Institute, Amsterdam, The Netherlands

**Keywords:** CD56^bright^ NK cells, Neurodegenerative diseases, Multiple sclerosis, Alzheimer’s disease, Parkinson’s disease, Amyotrophic lateral sclerosis

## Abstract

Emerging evidence suggests a potential role for natural killer (NK) cells in neurodegenerative diseases, such as multiple sclerosis, Alzheimer’s disease, Parkinson’s disease and amyotrophic lateral sclerosis. However, the precise function of NK cells in these diseases remains ambiguous. The existence of two NK cell subsets, CD56^bright^ and CD56^dim^ NK cells, complicates the understanding of the contribution of NK cells in neurodegeneration as their functions within the context of neurodegenerative diseases may differ significantly. CD56^bright^ NK cells are potent cytokine secretors and are considered more immunoregulatory and less terminally differentiated than their mostly cytotoxic CD56^dim^ counterparts. Hence, this review focusses on NK cells, specifically on CD56^bright^ NK cells, and their role in neurodegenerative diseases. Moreover, it explores the mechanisms underlying their ability to enter the central nervous system. By consolidating current knowledge, we aim to provide a comprehensive overview on the role of CD56^bright^ NK cells in neurodegenerative diseases. Elucidating their impact on neurodegeneration may have implications for future therapeutic interventions, potentially ameliorating disease pathogenesis.

## Background

Natural killer (NK) cells are a subset of innate lymphoid cells (ILCs) that represent an essential part of the innate immune system [1, 2]. NK cells are classified into two functionally and phenotypically distinct subsets based on their expression of CD16 and CD56: CD56^bright^CD16^−^ and CD56^dim^CD16^+^ NK cells, commonly referred to as CD56^bright^ and CD56^dim^ NK cells, respectively [3]. While CD56^bright^ NK cells are often considered to have an immunoregulatory role, their CD56^dim^ counterpart is thought to exert mainly cytotoxic functions.

NK cells are involved in various diseases of the central nervous system (CNS) and peripheral nervous system (PNS), although their exact role with respect to neuroprotection or neurotoxicity is still under debate. Evidence exists for both a beneficial or detrimental contribution of NK cells to the pathophysiological processes underlying neurodegenerative diseases, such as multiple sclerosis (MS), Alzheimer’s disease (AD), Parkinson’s disease (PD) and amyotrophic lateral sclerosis (ALS). For example, in MS, CD56^bright^ NK cells accumulate in periventricular brain areas, and a neuroprotective function has been proposed, possibly by inhibiting autoreactive T cells [4, 5]. Contrarily, in AD, the secretion of cytokines by NK cells correlates with a decline in cognitive abilities, but current studies do not differentiate between CD56^bright^ and CD56^dim^ NK cells [6, 7].

In this review, we provide an overview on the role of NK cells in health, non-neurodegenerative and neurodegenerative diseases. We describe how NK cells can migrate into the CNS, while focusing on the differences between CD56^bright^ and CD56^dim^ NK cells, and question the potential immunoregulatory role of CD56^bright^ NK cells. Accordingly, we also describe how the current therapies to treat neurodegenerative diseases are targeting or modifying NK cell functions.

## NK cells in health

### Origin and development of NK cells

**Fig. 1:**
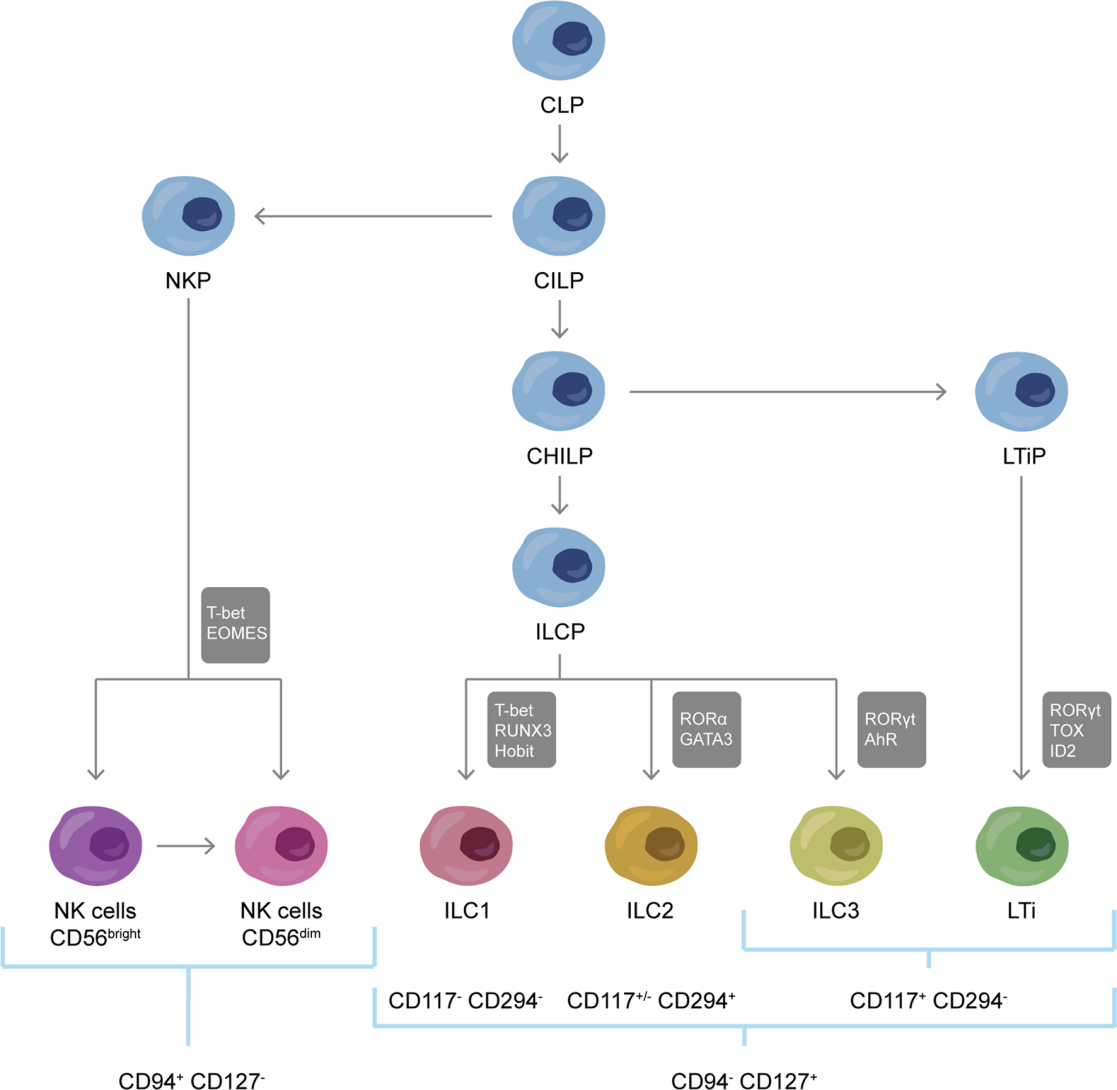
Innate lymphoid cells development. Common lymphoid progenitors (CLPs) differentiate into common innate lymphoid progenitors (CILPs), which can give rise to natural killer precursors (NKP) and common helper innate lymphoid progenitors (CHILPs) [19]. By expressing the transcription factors (TF) T-bet and EOMES, NKP cells can develop into NK cells. In turn, CHILPs can differentiate into innate lymphoid cell precursors (ILCPs) or lymphoid tissue inducer precursors cells (LTiP) [20]. ILC1s, ILC2s and ILC3s originate from ILCPs upon expression of different transcription factors (grey boxes). Finally, LTiP can generate LTi cells. To distinguish ILC subtypes, a population negative for lineage markers of other immune cells should be identified first (e.g., CD3, CD4, CD8, CD14, CD15, CD16, CD19, CD20, CD33, CD34, CD203c, and FcɛRI). Next, NK cells can be defined as CD56^+^ CD94^+^ CD127^−^ whereas ILCs are CD94^−^ and CD127^+^. The specific subtypes can be distinguished based on their expression of CD117 and CD294, as ILC1s are CD117^−^ CD294^−^, ILC2 are CD117^±^ CD294^+^, and ILC3s and LTi cells are CD117^+^ CD294^−^ [21].

NK cells are one of the five distinct groups of ILCs, a heterogeneous group of lymphocytes belonging to the innate immune system, together with ILC1s, ILC2s, ILC3s and lymphoid tissue inducer (LTi) cells. ILCs functionally resemble various T-cell subsets, especially with regards to cytokine production, although they lack antigen receptors rearranged via recombination activating genes (RAG) [1]. NK cells can be both circulatory or tissue-resident and resemble CD8^+^ cytotoxic T cells in their production of cytokines and cytotoxic molecules, while diverging in the mechanisms underlying target cell recognition [8]. ILC1s, ILC2s and ILC3s share similarities with CD4^+^ T helper (Th) 1, Th2 and Th17 cells respectively. They are generally considered tissue-resident, although they can also be found in the circulation in health and disease [9–14]. Finally, LTi cells mainly have a role in the formation of secondary lymphoid organs and populate tissues during embryonal development [1, 15–17]. The development of the distinct ILC subgroups depends on the expression of transcription factors previously described (Fig. 1) [1, 18–21].

### Activation of NK cells

NK cells can be activated by either a broad range of cytokines, such as IL-2, IL-12, IL-15 and IL-18, or upon recognition of target cells (e.g., infected, stressed, or cancerous cells) [22–24]. NK cells can recognize cells via two different mechanisms: (1) the so-called induced-self-recognition: activating receptors expressed on the NK cells (such as NKG2D and natural cytotoxicity receptors (NCRs) NKp30, NKp44 and NKp46) bind to stress-induced self-molecules that are usually absent on healthy cells (such as UL16-binding proteins), thereby triggering NK cell recognition [18, 25–27]. (2) NK cells also express MHC-specific inhibitory receptors, specifically CD94/NKG2A and certain killer-cell immunoglobulin-like receptors (KIRs), that prevent activation of NK cells upon binding to MHC class I molecules on the target cell. Upon downregulation of MHC class I, e.g., in virus-infected or cancerous cells, NK cells will be triggered by this missing-self-recognition. In this case, cytotoxicity will no longer be inhibited, leading to lysis of the target cell [18, 28, 29].

Cytotoxicity of the target cell by NK cells can be induced either via the death receptor pathway or the granule secretion pathway [30]. The death receptor pathway involves the expression of tumor necrosis factor (TNF) superfamily proteins on NK cells, namely: TNF, Fas ligand and TNF-related apoptosis-inducing ligand (TRAIL). Interaction of these death ligands on the NK cell surface with their corresponding receptor on the target cell leads to target apoptosis through the so-called extrinsic pathway which involves caspase 8 activation.

In the granule secretion pathway, NK cells release lytic granules that can enter the attached target cell either through perforin-induced pores in the plasma membrane or through endocytosis. This process leads to the intrinsic apoptosis pathway activation. Besides these two complementary cytotoxic pathways, NK cells are also potent producers of cytokines, such as interferon gamma (IFN-γ) and TNF-α [31, 32], suggesting that NK cells can also exert important immunomodulatory functions [33–35].

### CD56^bright^ and CD56^dim^ NK cells

In humans, as previously mentioned, NK cells can be roughly divided into CD56^bright^CD16^−^ and CD56^dim^CD16^+^ NK cells (from here on referred to as CD56^bright^ and CD56^dim^ NK cells [3]. Although they are generally considered two distinct subsets, they seem to merely represent different stages of NK maturation, as CD56^dim^ NK cells are more differentiated than CD56^bright^ NK cells, indicating that CD56^bright^ NK cells are less mature [36, 37]. This is supported by the finding that the telomeres of CD56^bright^ NK cells are longer and that they can acquire a CD56^dim^-like phenotype upon activation with cytokines, although no downregulation of CD56 itself was observed [38]. To date, even if Chan et al*.*, with an interesting series of experiments, proposed that NK cell differentiation from a CD56^bright^ to a CD56^dim^ phenotype could be induced by contact with fibroblasts [39], the exact mechanisms underlying this differentiation still remain poorly understood and seem to be multifactorial and complex.

In addition, a small proportion of the NK cells are CD56^bright^CD16^+^ NK cells, which are thought to represent a functional differentiation intermediate between the CD56^bright^CD16^−^ and CD56^dim^CD16^+^ cells [40]. Furthermore, a minor fraction of NK cells are CD56^dim^CD16^−^ NK cells. Their developmental relationship to the other subsets as well as their biological function has not yet been established [41]. Finally, NK cells can also lack the expression of CD56 entirely, although these cells have mainly been described in chronic viral infections [42]. This subset of NK cells does not seem to be developmentally related to the CD56^bright^ and CD56^dim^ NK cells, as they are functionally impaired, have longer telomeres than CD56^dim^ NK cells and lack end-stage differentiation markers [43].

Interestingly, CD56^bright^ and CD56^dim^ NK cells are differentially distributed throughout the human body [44–46]. CD56^bright^ NK cells primarily occupy secondary lymphoid tissues and various other tissues, such as the gut, uterus, liver and kidneys, whereas CD56^dim^ NK cells predominate the peripheral blood, bone marrow, spleen and lungs [3, 44]. This difference in localization of both subsets can be explained by various factors among which their differential expression of chemokine receptors, which will be discussed in the section *Migratory capacity of CD56*^*bright*^* NK cells* [46, 47].

#### Cytotoxic functions

**Fig. 2:**
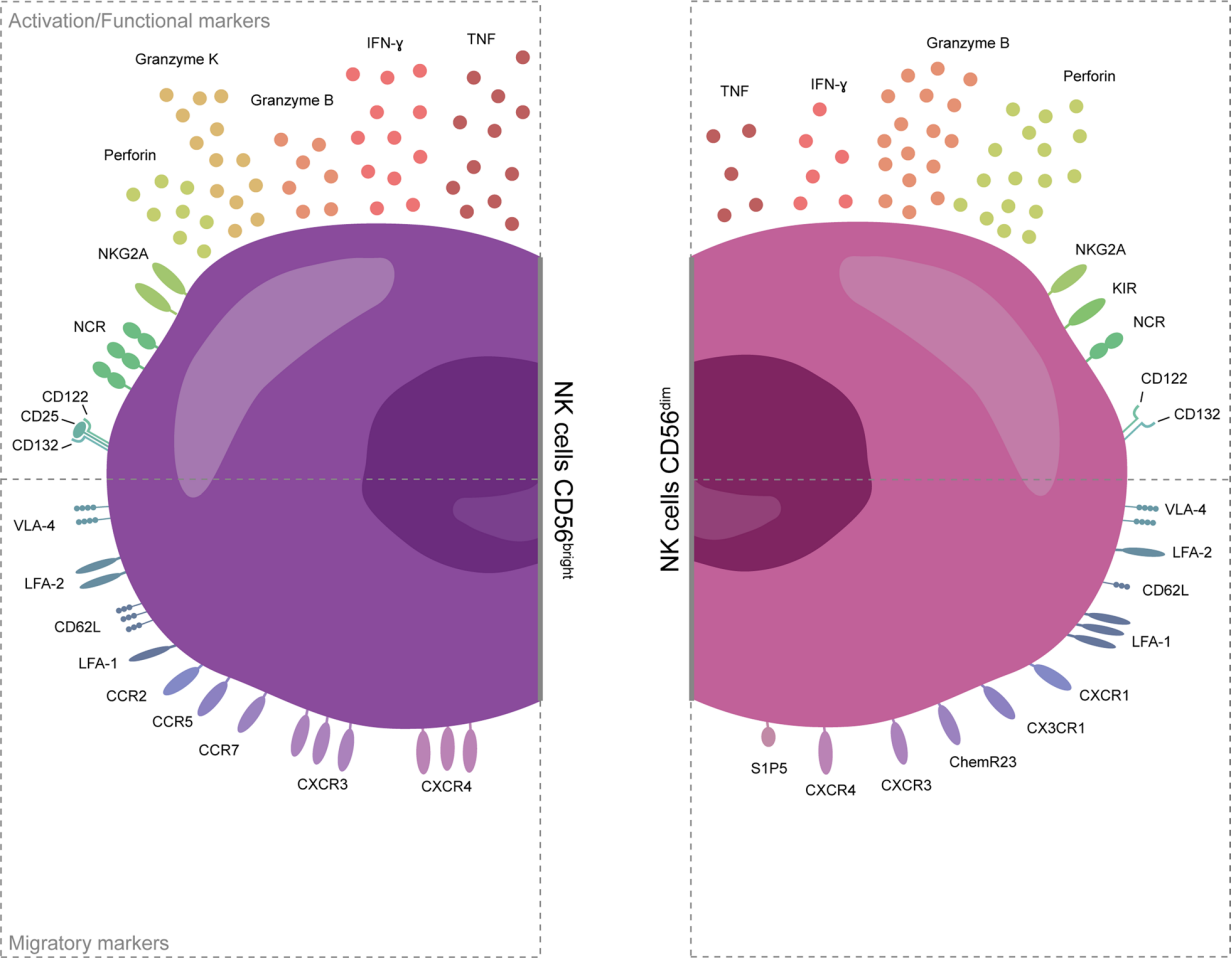
Differences in marker expression between CD56^bright^ NK cells and CD56^dim^ NK cells. Under homeostatic conditions, CD56^bright^ NK cells and CD56^dim^ NK cells express a variety of activation, functional and migratory markers that can be used to differentiate them, but also to explain their different locations and functions. In general, CD56^bright^ NK cells express higher levels of immunoregulatory proteins, such as tumor necrosis factor (TNF), interferon-γ (IFN-γ), granzyme K, natural killer group (NKG)2A, natural cytotoxicity receptors (NCR), and they express the trimeric high-affinity receptors for IL-2. In contrast, CD56^dim^ NK cells express the dimeric low-affinity receptor for IL-2 and higher levels of cytotoxic proteins, such as granzyme B and perforin. With regards to chemokine receptors, CD56^bright^ NK cells express C–C chemokine receptors (CCR)2, CCR5, CCR7, C–X–C motif chemokine receptor (CXCR)3, and CXCR4, while CD56^dim^ NK cells express CXCR1, CX3CR1, chemokine receptor (ChemR)23, and sphingosine-1-phosphate receptor 5 (S1P5), and low levels of CXCR3 and CXCR4. Furthermore, CD56^bright^ NK cells generally express higher levels of adhesion molecules lymphocyte function-associated antigen 2 (LFA-2) and CD62L (L-selectin), whereas CD56^dim^ NK cells preferentially express LFA-1 [59, 60, 65]. Both subsets express similar levels of very late antigen-4 (VLA-4) [61]. However, the expression of chemotactic receptors and adhesion molecules can be strongly affected by external stimuli

CD56^dim^ NK cells are thought to be more cytotoxic than their CD56^bright^ counterpart due to an increased expression of CD16, KIRs, granzymes and perforin (Fig. 2) [38, 44, 48, 49]. CD16, also known as Fcγ receptor III (FCγRIII) can bind the Fc region of immunoglobulins, leading to the activation of the NK cell and subsequent degranulation and secretion of perforin, resulting in the lysis of the target cell in a process called antibody-dependent cellular cytotoxicity (ADCC) [50, 51]. Although unstimulated CD56^bright^ NK cells express lower levels of these cytotoxic molecules compared to CD56^dim^ NK cells, upon activation, they are also capable of exhibiting cytotoxicity. For example, in vitro activation of CD56^bright^ NK cells with cytokines, such as IL-12 and IL-15, can induce degranulation and killing of autologous activated CD4^+^ T cells, mediated through the activating receptor NKG2D, lymphocyte function-associated antigen 1 (LFA-1) and the TRAIL pathway [5, 52, 53]. In addition, upon stimulation with IL-15, a large fraction of CD56^bright^ NK cells expresses granzyme B, similar to their CD56^dim^ counterpart [48]. These results are in line with the aforementioned observation that CD56^bright^ NK cells exhibit CD56^dim^-like features upon cytokine stimulation regardless of their unchanged CD56 expression [38]. Interestingly, in resting NK cells, the expression of granzyme K is significantly higher in CD56^bright^ NK cells than CD56^dim^ NK cells [40, 54], but upon stimulation with phorbol myristate acetate (PMA) and ionomycin the expression is down-regulated [55]. Granzyme B is known to induce target cell apoptosis by cleaving caspases and caspase pathway substrates, whereas granzyme K induces caspase-independent apoptosis by cleaving the nucleosome assembly protein SET [56, 57]. Taken together, these results indicate that CD56^dim^ NK cells are generally more cytotoxic than CD56^bright^ NK cells, although the cytotoxicity of both subsets is affected by the mechanisms through which the cells are stimulated [58].

#### Immunoregulatory functions

Similarly, specific methods of activation likely also affect the production of cytokines by each subset. In general, CD56^bright^ NK cells are more potent cytokine and chemokine producers, as they secrete high levels of cytokines, such as IFN-γ and TNF-α upon activation, thereby exerting immunoregulatory effects on both innate and adaptive immune cells (Fig. 2) [59–61]. Besides regulating the immune response through cytokine secretion, they can suppress autologous CD4^+^ T-cell activation via the activation of NCRs (NKp30, NKp44 and NKp46) and the secretion of granzyme K, resulting in mitochondrial dysfunction in activated T cells [5, 62]. Furthermore, studies suggest that activated CD56^bright^ NK cells in the lymph nodes may kill immature dendritic cells expressing low amounts of HLA-I molecules via activation of Nkp30 and secretion of IFN-γ in a process called DC editing, which ensures only the preservation of functionally mature dendritic cells [63, 64].

Indeed, in vitro intracellular cytokine production is highly dependent on the mechanism through which NK cells are activated. For example, a bigger fraction of the CD56^bright^ NK cells expresses intracellular IFN-γ upon stimulation with IL-12 and IL-18 compared to CD56^dim^ NK cells. However, activating the NK cells by co-culturing them with the tumor cell line K562 or cross-linking of activating receptors (such as NKp30, NKp46, CD2, NKG2D and 2B4) or CD16 results in a higher percentage of IFN-γ-expressing CD56^dim^ NK cells compared to CD56^bright^ NK cells [65]. Of note, only the percentage of intracellular IFN-γ^+^ cells and not the amount of IFN-γ production was quantified, and the duration of the stimulation varied across experiments. Although these results should, therefore, be interpreted with caution, they do demonstrate that both subsets respond differently to specific stimulations in terms of cytokine production.

Interestingly, CD56^bright^ NK cells respond more strongly to activation with soluble factors, such as cytokines, whereas CD56^dim^ NK cells are more responsive to cell surface ligands detected upon interaction with target cells [58]. This difference in the strength of response to certain stimuli can be explained by the expression of the corresponding receptors on both cell types [66]. For example, CD56^bright^ NK cells express the trimeric high-affinity receptors for IL-2 (CD25, CD122, CD132), whereas CD56^dim^ NK cells express only the dimeric low-affinity receptor β (CD122) and γ (CD132) chains, explaining why CD56^bright^ NK cells respond more strongly to IL-2 (Fig. 2) [60]. Therefore, the total production of cytokines and cytotoxic molecules by CD56^bright^ or CD56^dim^ NK cells depends not only on their cell type and abundance, but also on the method of stimulation. Taken together, these in vitro results suggest that the functional dichotomy between CD56^bright^ and CD56^dim^ NK cells, typically characterized as immunoregulatory and cytotoxic respectively, may not be as straightforward as commonly postulated. Instead, the properties of both subsets are likely strongly affected by the tissue- and disease-specific microenvironment.

### NK cells during aging

**Fig. 3:**
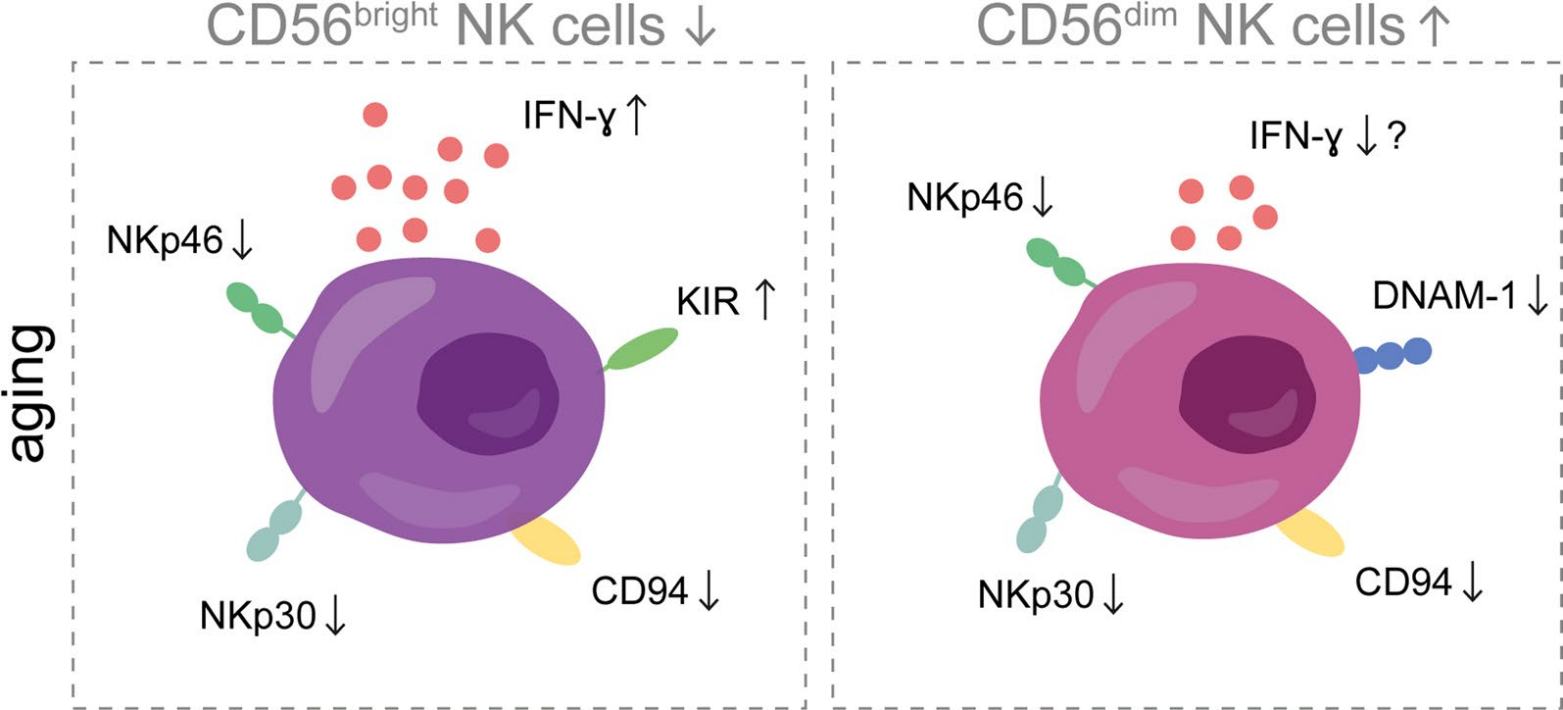
Differences between CD56^bright^ and CD56^dim^ NK cells during aging. During aging, CD56^bright^ NK cells are decreased in circulation while their counterpart CD56^dim^ NK cells increase their numbers in blood. Both in circulating CD56^bright^ and CD56^dim^ NK cells, the expression of the receptor CD94 and natural cytotoxicity receptors (NCRs) NKp30 and NKp46 is decreased in elderly subjects, whereas the expression of killer-cell immunoglobulin-like receptors (KIRs) seems to be upregulated only in CD56^bright^ NK cells. The expression of DNAM-1 seems to be decreased mostly in CD56^dim^ NK cells with age. These phenotypic changes might explain the decreased cytotoxicity observed during aging [67]

In line with these findings, the characteristics of the NK cell population are impacted by the process of aging. Although some discrepancies exist, most studies report an increase in the total number of circulating NK cells during aging [67, 68]. The composition of this population is altered, with a decrease in the percentage of CD56^bright^ NK cells and an increase in the percentage of CD56^dim^ NK cells [67]. Furthermore, following stimulation with IL-2 or IL-12, the production of certain chemokines and cytokines is decreased in the circulating NK cell population of healthy elderly compared to younger controls [67]. However, no distinction was made between CD56^bright^ and CD56^dim^ NK cells in these studies. Therefore, it remains unclear if NK cells in elderly secrete less cytokines and chemokines in response to stimulation or whether this effect may be caused by the decreased proportion of CD56^bright^ NK cells. The latter theory is supported by the fact that CD56^bright^ NK cells, but not CD56^dim^ NK cells, significantly increase the expression of IFN-γ in elderly individuals compared to younger individuals upon stimulation with IL-15 [69]. Besides the altered production of cytokines and chemokines, the expression of various NK cell receptors is affected in aging, as visualized in Fig. 3 [70–72]. These altered expression profiles might also underlie the reduction of NK cell cytotoxic activity that takes place in aging [67]. For example, the reduced expression of activating NCRs on both NK cell subsets, the increased expression of inhibitory KIRs on CD56^bright^ NKs, and the reduced expression of activating receptor DNAM-1 on CD56^dim^ NK cells might account for the hampered target cell recognition and killing in aged individuals. Finally, not only intracellular changes but also changes in the microenvironment may affect the functionality of NK cells in aging, such as the decreased levels of IL-2 and IL-15 known to be vital for maintaining NK cell function [67].

### Murine NK cells

As murine NK cells do not express CD56, they cannot be directly compared to the human CD56^bright^ and CD56^dim^ subsets, although they can be divided into two subsets based on the expression of CD27 [73, 74]. Unbiased clustering of the transcriptomic profiles of NK cells in the spleen and blood of mice and brain-dead human donors validated the correspondence of the murine CD27^high^ and CD27^low^ to human CD56^bright^ and CD56^dim^ NK cells, respectively [75]. Similar to the human CD56^bright^ NK cells, murine CD27^high^ NK cells mature into CD27^low^ NK cells [20, 75, 76]. Furthermore, these murine CD27 and human CD56 subsets share similarities with regards to the chemokine receptor expression and distribution across tissues, as for example murine CD27^low^ NK cells predominate in the peripheral blood and lungs and murine CD27^high^ NK cells are the main NK population in the lymph nodes [46, 74]. However, a large population of murine CD27^high^ NK cells has also been observed in the bone marrow, whereas in humans mostly CD56^dim^ NK cells reside in the bone marrow, demonstrating that these subsets cannot be compared directly [3, 74]. With regards to functional properties, the murine CD27^high^ NK cells secrete higher levels of IFN-γ upon stimulation with IL-12 and IL-18 compared to murine CD27^low^ NK cells, further supporting their similarity to human CD56^bright^ NK cells [74]. In line with this, murine CD27^low^ NK cells are more strongly associated with cytotoxicity [76, 77]. Strikingly, CD27^low^ and CD27^high^ subpopulations can also be identified in humans. Similar to the murine CD27 subsets, human CD27^high^ NK cells are potent cytokine producers, whereas human CD27^low^ NK cells are more cytotoxic [73, 78]. In peripheral blood, most NK cells are CD56^dim^ CD27^low^ and a small percentage is CD56^bright^ CD27^high^, although populations expressing CD56^bright^ CD27^low^ and CD56^dim^ CD27^high^ were also observed [79]. Therefore, despite the existence of numerous similarities between the murine CD27 and human CD56 subsets, caution should be exercised when comparing studies on these human and murine NK cell subsets.

## Migratory capacity of CD56^bright^ NK cells

A crucial mechanism underlying the function of CD56^bright^ NK cells is their migration into peripheral tissues. In general, leukocytes migrate from their tissue of origin into the blood or lymphatic vasculature upon maturation as a result of differential expression of chemokine receptors and signaling molecules [80]. Here, they circulate until migrating into peripheral tissue in response to inflammatory stimuli or to patrol for possible pathogens [80]. This migration can be modulated by the presence of cytokines, chemokines and cellular adhesion molecules (CAMs) in the tissue and the expression of the corresponding receptors on the leukocytes, a process that has been extensively reviewed previously [80]. Briefly, endothelial cells become activated by inflammatory stimuli, resulting in their upregulation of CAMs (such as the selectins E-selectin and P-selectin). Low affinity binding between these selectins and leukocyte glycoproteins [e.g., P-selectin glycoprotein ligand-1 (PSGL-1)] facilitates the capture, adhesion and rolling of leukocytes over the endothelial cells. Next, integrins such as LFA-1 and very late activation antigen-4 (VLA-4) expressed on leukocytes bind to intercellular adhesion molecule 1 (ICAM-1 or CD54) and vascular cell adhesion molecule 1 (VCAM-1 or CD106) on the endothelial cells, respectively, strengthening the bond between both cell types, and eventually leading to diapedesis of the leukocytes through the endothelium [81–83].

In this review, we will describe the current knowledge regarding the migration of CD56^bright^ NK cells towards peripheral tissues and especially towards the CNS. By elucidating the mechanisms underlying the migration of NK cells into the CNS, we may gain valuable insights into their potential function in neurodegenerative disorders and possible therapeutic interventions targeting this migration.

### Migratory markers on NK cells

CD56^bright^ and CD56^dim^ NK cells differentially express chemokine receptors and adhesion molecules, such as integrins (described in Fig. 2), explaining their distinctive migration from blood towards various peripheral tissues as a result of tissue-specific expression of ligands corresponding to these receptors [46, 47, 59–61, 84, 85]. For example, the increased expression of C–C chemokine receptor type 7 (CCR7) on CD56^bright^ NK cells compared to CD56^dim^ NK cells explains the accumulation of CD56^bright^ NK cells in lymph nodes, where high levels of its ligands chemokine C–C motif ligand CCL19 and CCL21 are present [86]. Notably, the expression levels of chemotactic receptors and adhesion molecules on NK cells are strongly affected by environmental signals, such as inflammatory stimuli [87]. For instance, under homeostatic conditions, CD56^bright^ NK cells express higher levels of CD62L (known as L-selectin) than CD56^dim^ NK cells, but upon in vitro stimulation with IL-15, the expression of CD62L on CD56^bright^ NK cells is downregulated to a greater extent than on CD56^dim^ NK cells [55, 88]. Therefore, the migration of CD56^bright^ and CD56^dim^ NK cells is not only affected by their differential expression of adhesion molecules and chemotactic receptors, but also by the presence of environmental factors, such as pro-inflammatory cytokines.

### Migration towards the CNS

#### CNS barriers

**Fig. 4:**
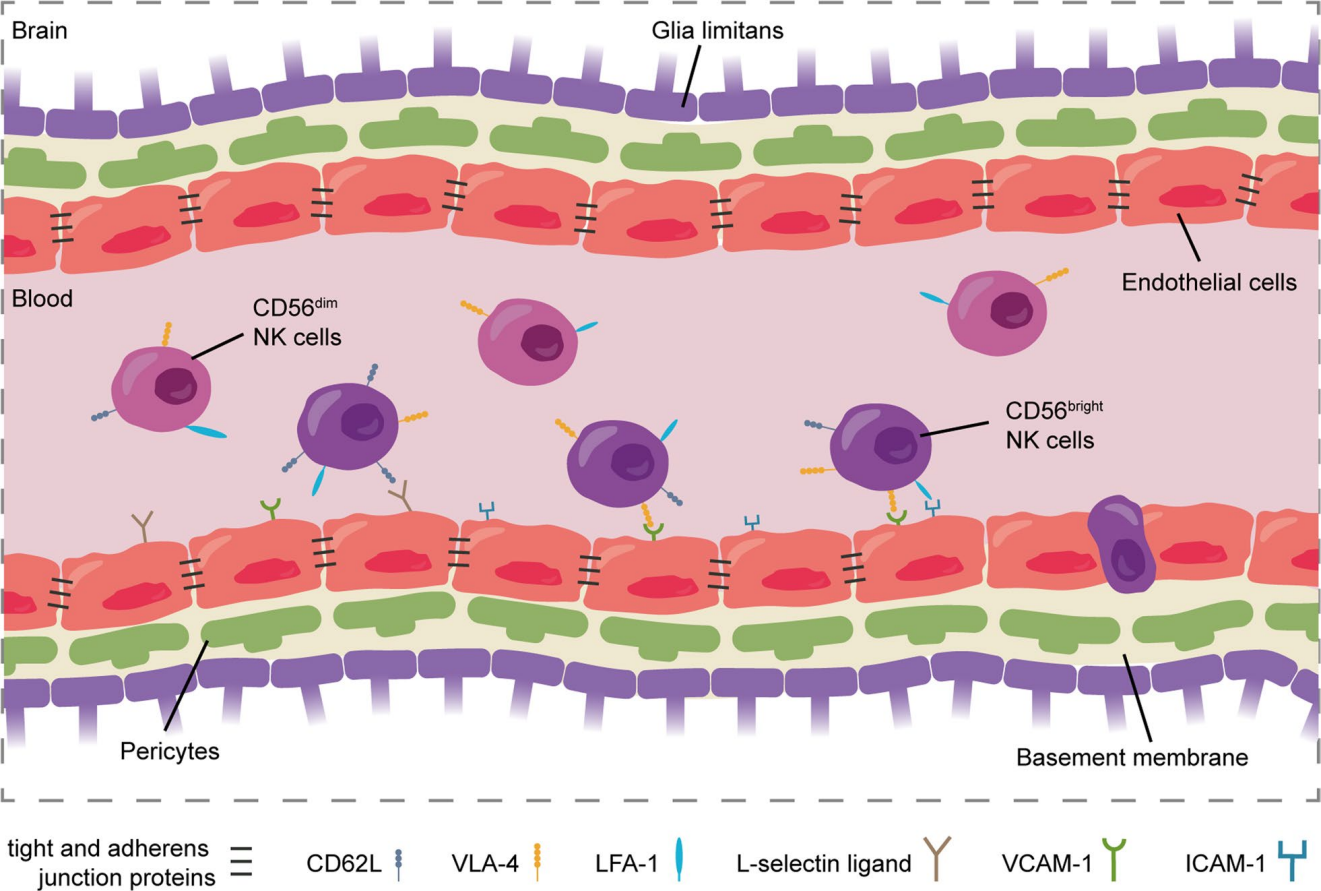
Migration of CD56^bright^ and CD56^dim^ NK cells across the blood–brain barrier. The blood–brain barrier (BBB) is formed by the closely connected brain endothelial cells that line the vessels, their basement membrane, pericytes and the astrocytic end-feet or glia limitans [89]. While the exact molecular interactions that allow NK cell migration across the BBB are currently unknown, a small proportion of NK cells can cross this barrier under unstimulated conditions, with CD56^bright^ NK cells migrating towards the brain in higher numbers that CD56^dim^ NK cells [61]

The CNS is protected from the entry of toxins and pathogens via the blood–brain barrier (BBB) [89]. The BBB is composed of brain endothelial cells that line the vessels and are closely connected by tight junction and adherens junction proteins [90, 91]. Barrier properties of the endothelial cells are further enhanced by pericytes extending their cellular processes over the endothelial surface, microglial cells contacting endothelial cells and the astrocyte endfeet surrounding the brain capillaries [89, 92, 93]. Altogether, these connections form the neurovascular unit (Fig. 4).

Another brain barrier is the blood–cerebrospinal fluid barrier (BCSFB), which is formed by the epithelial cells lining the choroid plexus [94]. Besides acting as the main producer of cerebrospinal fluid (CSF), this highly vascularized tissue facilitates migration of peripheral immune cells into the CSF, thereby serving as an immunological niche enabling immunosurveillance [81, 95, 96]. The tight junctions, adherent junctions and gap junctions between the epithelial cells are essential for the regulation of the permeability, integrity and polarity of the BCSFB [94].

Finally, the blood–meningeal barrier (BMB) is also considered one of the CNS barriers. The meninges are composed of three distinct layers—namely, the dura mater, the arachnoid mater and the pia mater. Besides serving as a physically protective membrane for the underlying neural tissue, they also form an immunological niche by regulating the migration of immune cells into the CSF [81, 96]. The BMB is formed by the endothelial cells surrounding the vessels that are present in the subarachnoid space and infiltrate into the brain parenchyma [81, 96].

#### NK cell migration towards the CNS

Although not much is known regarding the presence of NK cells in the human brain parenchyma, NK cells have been identified in healthy mouse brain parenchyma, the majority of which were immature CD27^high^ cells [97, 98]. To date, it remains unclear how NK cells could traffic into the CNS. Using human brain microvascular endothelial cells (HBMECs) as a BBB model in vitro, a small proportion of NK cells of healthy donors were able to cross the BBB in unstimulated conditions [61]. This suggested that NK cells could indeed be present in the healthy human brain by trafficking across the BBB. In particular, mainly CD56^bright^ NK cells were capable of crossing this BBB model (Fig. 4) [61]. This corresponded with the previous findings that a CD27^high^ NK cell population is present in the murine brain, that CD56^bright^ NK cells predominate most peripheral tissues, and that they have an increased expression of certain migration markers such as CD62L compared to CD56^dim^ NK cells [46, 47, 61, 98]. Furthermore, granzyme K, secreted by CD56^bright^ NK cells, might facilitate migration by indirectly inducing ICAM-1 expression on endothelial cells, a process that has been shown to induce migration of granzyme K-producing effector memory CCR5^high^ T cells over unstimulated HBMECs [99].

NK cells are also present in the choroid plexus and to a lesser extent in septum of healthy donors [4]. Since the septum is also highly exposed to CSF, these results suggest another possible route of migration via the BCSFB. Corresponding to these findings, multiple studies have identified a small population of NK cells in the CSF [61, 100–103]. Although we cannot exclude that these cells arrive to the CSF via the BMB and not via the BCSFB, limited knowledge exists regarding the presence of NK cells in the meninges. One recent study shows NK cells in the human leptomeninges via single-nuclei RNA sequencing, and one other study has identified NK cells in the murine meninges, and both studies did not distinguish between the different NK cell subsets [104, 105]. Finally, inconsistent with the presence of mostly immature NK cells in mouse brain parenchyma, the human choroid plexus seem to consist mostly of CD56^dim^ NK cells, although this might also be a result of the leakage of blood into the tissue [4]. Therefore, more research is required to validate the presence of NK cell subsets in the healthy human brain and the possible routes of trafficking during homeostasis.

Under inflammatory conditions, the expression of integrins on leukocytes and adhesion molecules on the BBB is upregulated, and the affinity of integrins for adhesion molecules is increased as a result of conformational changes, together facilitating the migration of leukocytes over the BBB [81, 106]. In vitro, a higher percentage of NK cells from healthy donors migrated over the aforementioned BBB model when the HBMECs were stimulated with IFN-γ and TNF-α [61]. Similarly, the barrier properties of the BCSFB are disturbed during neuroinflammation and chemokine production by the epithelial cells is increased, facilitating migration of leukocytes over the BCSFB, although this has mostly been studied using animal models [106–109]. However, to our knowledge, no research has been done defining the migration processes of NK cells across BCSFB models.

Finally, neuroinflammation is also associated with a dysfunctional BMB and an increase of immune cells in the BMB [4, 110, 111]. As previously mentioned, only two studies identified NK cells present in the meninges of both humans and mice, but these studies did not determine migration of these NK cells over the BMB [104, 105]. Since the barrier properties and the expression of cellular adhesion molecules of each brain barrier is differentially affected by the various neurodegenerative conditions, the migration of NK cells across the different barriers may depend on disease-specific characteristics. For these reasons, the migratory capacities of NK cells with regards to different brain barriers should be studied in the context of specific neurodegenerative diseases.

## CD56^bright^ NK cells in neurodegenerative diseases

### Multiple sclerosis

MS is an inflammatory demyelinating disease of the CNS that primarily affects young adults and it is characterized by gradually declining motor abilities and cognitive impairment [112]. There are multiple forms of MS, of which the relapsing–remitting type (RRMS) is the most prevalent. RRMS is characterized by stable periods without clinical decline, interrupted by episodes of inflammatory relapses. This irregular disease form is often considered the first stage of MS that is followed by a more gradual and progressive disease course, termed secondary progressive MS (SPMS). In addition, 5—15% of the patients have a progressive disease course from onset, a form of MS that was coined as primary progressive MS (PPMS). One of the pathological hallmarks of MS is the formation of demyelinating lesions in the CNS. Autoreactive immune cells can infiltrate the CNS leading to loss of myelin and consequent axonal loss and neurodegeneration [113]. In MS, different types of lesions can be distinguished based on their inflammatory status and location. Active lesions are characterized by focal immune infiltration, which promotes demyelination in the core of the lesion. Upon prolonged inflammation or progression, active lesions become chronic active lesions, where inflammation is mainly present at the rim of the lesion. Over time, these can develop into chronic inactive lesions, in which both the core and rim of the lesion lack immune cells [112]. Although MS is generally considered a disease of the adaptive immune system mediated by T cells and B cells, mounting evidence indicates a role for various cells of the innate immune system [114], such as the brain-resident microglia and the peripherally recruited macrophages [115]. In addition, innate-like B and T cells, such as B1 and γδ T cells, and ILCs, are currently under investigation for their possible involvement in autoimmune diseases, such as MS [116–118].

In vivo research on MS is commonly performed in an animal model called experimental autoimmune encephalomyelitis (EAE), in which inflammation of the CNS is induced via the administration of an adjuvant and a CNS antigen (e.g., myelin–oligodendrocyte glycoprotein (MOG), myelin proteolipid protein (PLP), or myelin basic protein (MBP)) or myelin-reactive T cells [119]. Several EAE studies showed that CD27^low^ NK cells, but not CD27^high^ NK cells, exacerbated the severity of EAE, suggesting that CD56^dim^ NK cells could be pathogenic in MS [120]. However, although in many patient studies an involvement of NK cells in MS pathogenesis has been proposed, knowledge regarding the role of the specific CD56^bright^ and CD56^dim^ subsets is lacking. In this review, we will summarize the main findings regarding the involvement of CD56^bright^ NK cells in human MS patients and we will review the current knowledge on NK cells in EAE and if those functions could be attributed to the possible CD56^bright^ NK cells in humans.

#### Location and migration

CD56^bright^ NK cells are thought to predominate the CNS, while their more cytotoxic CD56^dim^ counterpart is the primary subset in the peripheral blood [4, 61]. In general, the proportion of CD56^bright^ NK cells to the total NK cells in peripheral blood decreases upon age, a phenomenon that is even more pronounced in PPMS [49, 121]. Therefore, although multiple studies have observed a similar percentage of peripheral CD56^bright^ NK cells in controls and MS [49, 53], this may depend on the age of the study subjects or the type of MS. In the brain of MS patients, CD56^bright^ NK cells accumulate in periventricular areas, such as the septum [4]. Furthermore, NK cells expressing granzyme K, which is mainly expressed by CD56^bright^ NK cells [4, 5], are present in MS lesions and even polarized towards T cells [61]. This suggests that in MS, CD56^bright^ NK cells might interact with other immune cells in the brain and consequently modify the local immune responses.

**Fig. 5:**
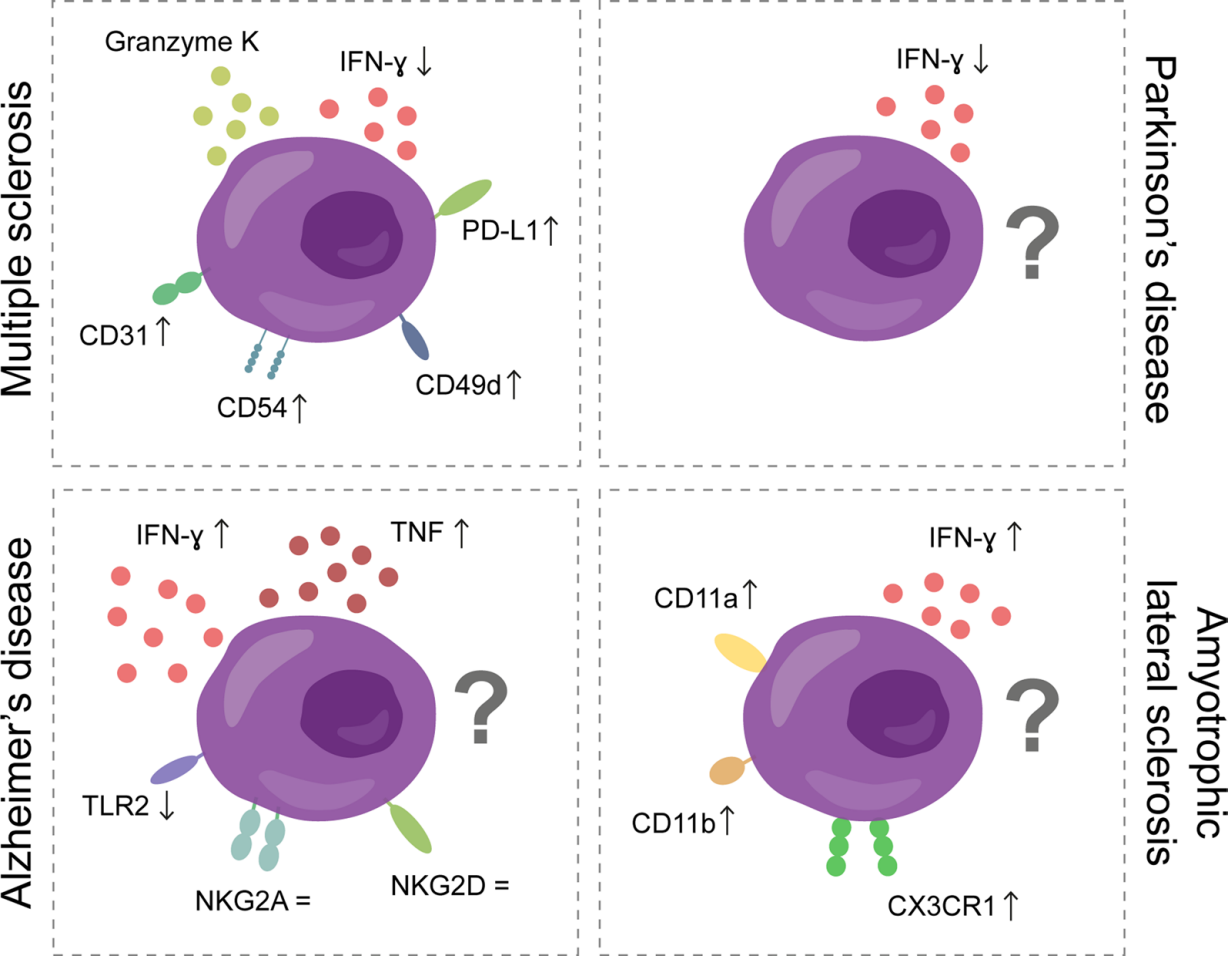
CD56^bright^ NK cells in multiple sclerosis (MS), Alzheimer’s disease (AD), Parkinson’s disease (PD) and amyotrophic lateral sclerosis (ALS). CD56^bright^ NK cells accumulate in periventricular areas of MS patients, where they can secrete granzyme K in close contact of T cells. They also express granzymes and migratory markers in higher levels compared to control donors, which could explain their propensity towards the brain [4, 5]. In PD, AD and ALS, more research is available on NK cells, without CD56^bright^ and CD56^dim^ distinction. Similarly as in MS, NK cells from PD patients release lower levels of IFN-γ compared to controls. In contrast, NK cells release a plethora of pro-inflammatory cytokines in AD and ALS, probably explaining their pathogenic potential in those diseases [6, 216, 230]

The accumulation of CD56^bright^ NK cells in the CNS might be due to increased migration across the CNS barriers. As already mentioned, CD56^bright^ NK cells have a stronger adhesion and higher migratory capacity across a human model of the BBB in vitro, compared to CD56^dim^ NK cells. Interestingly, the increased migration of CD56^bright^ NK cells is irrespective of the disease status: no differences were found between CD56^bright^ NK cells derived from healthy donors or MS patients [61], although peripheral CD56^bright^ NK cells in MS patients express higher levels of the CAMs CD49d (subunit of VLA-4) and CD31 compared to controls (Fig. 5**)** [4]. This indicates that this transmigration process can depend on other CAMs. However, this in vitro model does not perfectly replicate the in vivo situation, as for example no chemokines were present and the NK cells were not activated before performing the migration assay. In addition, higher numbers of CD56^bright^ NK cells compared to CD56^dim^ NK cells are found in the CSF of MS patients [4], which also suggests that this subset might migrate more across the BMB and the BCSFB.

#### Function

Because of their immunomodulatory properties, the CD56^bright^ subset is suggested to have beneficial effects on the MS disease course [116, 122], although contradictory findings have been reported in both MS and EAE. Another possibility is that these immunomodulatory properties might be defective during MS, thereby preventing the ability of NK cells to suppress the proliferation of autoreactive T cells.

CD56^bright^ NK cells can kill activated autologous T cells in a granzyme K-dependent manner, by inducing apoptosis of the target cells through mitochondrial dysfunction, leading to reactive oxygen species (ROS) production and subsequent cell death [5, 30, 123, 124]. This killing was enhanced in NK cells derived from daclizumab-treated MS patients. Daclizumab is a monoclonal antibody against the α-chain of IL-2R (IL-2Rα or CD25) and is thought to enhance NK-mediated killing of autoreactive T cells by expanding CD56^bright^ NK cells and increasing their granzyme K expression [5, 61, 100, 123, 124] (*more information in the subsection of Therapies*). Furthermore, CD56^bright^ NK cells in the brain of MS donors had increased expression of the checkpoint inhibitor programmed death-ligand 1 (PD-L1) [4], which can bind to programmed cell death protein 1 (PD-1) on T cells and induce the suppression of autoreactive T cells [125]. In addition, MS often ameliorates during pregnancy, in which CD56^bright^ NK cells increase in the circulation of pregnant women. It is, therefore, hypothesized that this subset contributes to the control of inflammation during pregnancy [126].

As mentioned previously, CD56^bright^ NK cells are capable of killing autologous T cells via granzyme K. However, CD56^bright^ NK cells from untreated MS patients have an impaired killing of activated autologous T cells in vitro. This defective immunomodulatory function might be due to the increased expression of HLA-E by T cells in MS, which binds to the inhibitory NK receptor NKG2A, thereby inhibiting the cytotoxicity of CD56^bright^ NK cells [53]. In addition, HLA-E expression is upregulated in white matter lesions in MS, specifically on endothelial cells and astrocytes, but also on microglia and infiltrating T cells [127, 128]. This could indicate that CNS brain cells, such as endothelial cells, astrocytes or microglia, can impair the immunomodulatory capacity of CD56^bright^ NK cells in MS. Furthermore, soluble HLA-E is also increased in serum and CSF of RRMS patients. Interestingly, CSF samples from MS patients that contained soluble HLA-E were more effective in inhibiting NK cell cytotoxicity in vitro [128]. Hence, CD56^bright^ NK cells might have the ability to suppress inflammation in the brain and periphery during MS, but their function might be inhibited upon interaction with other immune cells or even CNS cells.

Finally, CD56^bright^ NK cells are known for the large secretion of various cytokines, such as IFN-γ [129]. This cytokine plays a deleterious role in MS, it is increased during MS activity and its blockage is beneficial for MS patients [130, 131]. CD56^bright^ NK cells from RRMS patients have decreased production of IFN-γ compared to healthy donors [132, 133], which also suggests that the impairment of CD56^bright^ NK cells in MS might be advantageous for the patient.

In animal research, there are even more contradictory results on whether NK cells have a protective or pathogenic role during EAE, possibly due to the lack of CD56 expression on murine NK cells. Although the aforementioned CD27 is often used to distinguish similar NK cell subsets in mice, most studies on NK cell function in EAE did not look into the separate contribution of these subsets.

Depletion of all NK cells before EAE induction, using an anti-NK1.1 antibody, leads to worsened disease course [134, 135]. This suggests that NK cells have immunomodulatory effects in EAE, but results should be interpreted with caution since NK1.1 is also expressed by ILC1s, ILC3s and T cells [136]. In contrast, a similar study where the authors depleted NK cells using an anti-NK1.1 antibody or an anti-asialo GM1 antibody led to earlier disease onset, but improved clinical symptoms in EAE [137]. However, as GM1 is also expressed by T cells [136, 138], it is likely that the studies using these depletion methods did not exclusively deplete NK cells.

Other studies indicating a protective role of NK cells in EAE also suggested the importance of the interaction between the chemokine CX3CL1 and its receptor CX3CR1 expressed on NK cells. During neuroinflammation, neurons can secrete CX3CL1, resulting in NK cell migration towards the inflamed CNS [139, 140]. In line with this, CX3CR1-deficient mice with EAE have reduced quantities of NK cells in the CNS, indicating a lack of NK cell migration towards the CNS. These animals have exacerbated EAE pathology and disease course. In addition, transplantation of CX3CR1^+^ NK cells into the CX3CR1-deficient mice delayed EAE onset, incidence and disease severity [135, 140]. However, we must remark that in humans CX3CR1 is mainly expressed by CD56^dim^ NK cells, thereby, complicating the translation of these results to humans [141].

Another mechanism through which NK cells might regulate the immune response in the CNS of EAE animals is via the expression of choline acetyltransferase (ChAT). This enzyme synthesizes the neurotransmitter acetylcholine, which is known to have anti-inflammatory properties [142]. ChAT expression in NK cells is increased upon their maturation and is further enhanced after EAE induction [142, 143]. Transplantation of CX3CR1^+^ ChAT^+^ NK cells into CX3CR1-deficient mice also diminished the severity of the symptoms in EAE [143]. These results also suggest that the maturation of NK cells, resulting in increased ChAT expression, may be an anti-inflammatory compensatory mechanism in EAE. One could speculate that this would mean increased ChAT expression in CD56^dim^ NK cells in humans. However, the upregulation of ChAT expression is only found in CD56^bright^ NK cells of MS patients [143].

As previously mentioned, there are contradictory results regarding the role of NK cells. Other studies suggested a detrimental role of NK cells during EAE. For example, in an EAE model, in the B6 mouse strain, neural stem cells of the subventricular zone secreted IL-15, which recruited and retained NK cells in this zone during the chronic phase of EAE. Subsequently, these NK cells killed neural stem cells due to the downregulation of Qa1 on the neuronal stem cells, a murine MHCI molecule that binds the inhibitory NKG2A receptor on NK cells. By depleting these cells, NK cells inhibited oligodendrogenesis and myelin injury recovery [144].

Both human and EAE research show that NK cells are, directly or indirectly, involved in the pathogenesis of MS. Although the exact mechanisms underlying a possible beneficial role for CD56^bright^ NK cells in MS remain controversial, it is clear that these cells interact with CNS cells or other immune cells. Furthermore, it has been established that different therapies, especially daclizumab, affect NK cells in MS. Therefore, in the next section we describe the current knowledge regarding MS therapies and their effect on CD56^bright^ NK cells.

#### Therapies

To date, there is no cure available for MS, but there is an armament of different disease-modifying therapies (DMTs) that might alleviate the disease burden. The majority of these therapies target the immune responses and are successful in treating RRMS patients but they have limited efficacy in patients with the progressive forms of the disease [145–147].

Daclizumab was designed as an immunosuppressive drug targeting autoreactive effector T cells. Upon activation, effector T cells upregulate the expression of IL-2Rα (CD25), which increases the affinity of IL-2R for its ligand, the cytokine IL-2 [124, 148, 149]. Daclizumab blocks the interaction of IL-2 and the activated T cells that express high-affinity IL-2R, thereby inhibiting the proliferation and activation of these cells. Concomitantly, IL-2 is increasingly available to bind to cells that also express intermediate-affinity IL-2R, such as CD56^bright^ NK cells, possibly resulting in their activation and expansion [123, 150]. CD56^bright^ NK cells express this receptor in higher levels than CD56^dim^ NK cells, suggesting that daclizumab has a stronger effect on CD56^bright^ NK cells than CD56^dim^ NK cells. Moreover, daclizumab may inhibit the trans-presentation of IL-2 and IL-15 to naïve T cells by dendritic cells, deemed essential for the development of antigen-specific T cells [151]. At the same time, this may increase IL-15 availability, which also activates CD56^bright^ NK cells. Finally, daclizumab might increase CD155 expression on T cells and thereby restore the interaction between the activating receptor DNAX Accessory Molecule-1 (DNAM-1/CD226) on NK cells and CD155 on T cells. This is considered to be a prerequisite for NK-mediated immunoregulation [61]. Thus, the beneficial effects of daclizumab in MS are likely partially mediated by an immunoregulatory function of NK cells. However, daclizumab was withdrawn from the market worldwide in 2018 [152]. While MS patients treated with daclizumab had a reduction of new lesions in the brain and had improvements in physical and psychological functioning, 84% of the patients suffered from adverse events with 16% of the patients having mild or moderate hepatic toxicity, as well as secondary CNS-autoimmunity with encephalitis and/or meningitis [153, 154]. After one fatal case of liver failure and four cases of serious liver injuries, daclizumab was suspended from the market [155–157]. The mechanisms behind this hepatic toxicity are not yet totally understood, but it has been suggested that hyperactivation of CD56^bright^ NK cells might lead to enhanced cytotoxic function and increased migration of this subset into the liver [158]. All in all, this raises the awareness that the fine-tuning response of CD56^bright^ and CD56^dim^ NK cells is important to study to understand how these cells behave and how they might be targeted by different therapies.

Daclizumab was the first MS therapy that showed an effect on CD56^bright^ NK cells and since then, more research has been done to study the effect of various therapies on CD56^bright^ NK cells (Table 1). Some of these therapies resulted in the proliferation of CD56^bright^ NK cells, which correlated with positive treatment outcomes, while other therapies modified the activation of these cells, but without leading to hyperactivation. However, the majority of studies did not differentiate between CD56^bright^ NK cells and CD56^dim^ NK cells, which complicates interpretation of the data and might explain the contradictory results shown in MS research regarding the role of NK cells. Nonetheless, studies using human MS material suggest a beneficial immunomodulatory role for the CD56^bright^ NK cell population in the pathogenesis of MS.

**Table 1:** MS therapies and their effect on CD56^bright^ NK cells

Therapy	Mode of action	Effect in blood	Effect in CNS	References
Daclizumab	Monoclonal antibody against the α-chain of IL-2Rα. Inhibits effector T-cell activation, regulatory T-cell expansion and activation-induced T-cell apoptosis	Increases CD56^bright^ NK cell numbers via IL-2Rβ intracellular signaling	Increases CD56^bright^ NK cells in the CSF	[5, 61, 100, 123]
Natalizumab	Monoclonal antibody against α4-integrin of VLA-4. Blocks the binding of immune cells to endothelial cells and prevents immune cell migration towards the CNS	Increases total NK cell numbers. Decrease frequency CD56^bright^ NK cells	Blocks trafficking of NK cells. Decreases numbers of NK cells in the CNS and lymph nodes	[159–161]
Interferon-β	Immunomodulatory properties. Inhibits T-cell proliferation and impairs trafficking of immune cells into the CNS	Expands CD56^bright^ NK cells and even higher expansion on responders. Alters the phenotype with decreased expression of the inhibitory receptor LILRB1 and increased expression of NKG2A receptor	NA	[162–165]
Glatiramer acetate	Resembles myelin basic protein and binds to MHCII in antigen-presenting cells, competing with other antigens and limiting the presentation of self-antigens	No differences of CD56^bright^ NK cell frequencies. Enhances killing of autologous immature and mature dendritic cells in vitro and in MS patients. Increases expression of activating NKG2D and NKp46 receptors	No differences in NK cell numbers nor CD69 expression	[166–170]
Fingolimod	Agonist of sphingosine 1-phosphate receptor, which inhibits lymphocyte egress from lymph nodes, preventing the migration of autoreactive T cells into the CNS	Decreases CD56^bright^ NK cell numbers, and even higher decrease in non-responders. No effects on cytokine production of IFN-γ, TNF and IL-10, nor in HLA-DR expression on NK cells. Increases migration in vitro towards CXCL12, but decreased towards CX3CL1. Blocks the egress of immature NK cells from the lymph nodes	NA	[160, 171–174]
Dimethyl fumarate	Fumaric acid ester that activates the nuclear factor-kβ pathway and the neuroprotective nuclear factor (erythroid-derived 2)-related factor 2, which regulates inflammation and protects neural cells	Increases the number of CD56^bright^ NK cells. This increase correlated negatively with the numbers of CD8^+^ T cells producing IFN-γ and TNF. Increases numbers of CD107a^+^ cells, as a measure of increased degranulation	NA	[175–177]
Ocrelizumab	Humanized monoclonal antibody that targets CD20^+^ cells	Discrete decrease of CD56^bright^ NK cells	NA	[178]
Cladribine	Synthetic chlorinated deoxyadenosine analogue. Cladribine phosphates accumulate in proliferative cells interfering with DNA synthesis and repair, which leads to cell death. The main effects are in lymphocytes	No effects on CD56^bright^ NK cell numbers	NA	[14, 179]

CNS: central nervous system; CSF: cerebrospinal fluid; DNA: deoxyribonucleic acid; IL-2R: interleukin-2 receptor; IFN: interferon; LILRB1: Leukocyte immunoglobulin-like receptor subfamily B member 1; MHCII: major histocompatibility complex II; MS: multiple sclerosis; NA: not available; NK: natural killer; NKG2A: natural killer group 2A; NKG2D: natural killer group 2D; TNF: Tumor necrosis factor; VLA-4: very late antigen-4

### Alzheimer’s disease

AD is the most common form of dementia and is associated with loss of memory and speech and cognitive decline [180]. There are two types of AD based on the age of onset and the genetic predisposition. Early onset AD or familial AD occurs before the age of 60–65 and is driven by mutations in amyloid precursor protein (APP) or presenilin 1 or 2 (PS1–2) genes, which leads to an overproduction of amyloid-β (Aβ) peptides in the brain [81]. The most frequent form of AD is sporadic or late-onset AD, which usually begins after the age of 65 years and is associated with various environmental and genetic risk factors, such as carrying the apolipoprotein ε4 allele (APOE4) [181, 182]. Both forms of AD exhibit similar pathological hallmarks, such as extracellular deposits of Aβ peptides and aggregation of hyperphosphorylated tau protein into neurofibrillary tangles inside neurons. Furthermore, neurovascular function is decreased, leading to cerebral brain hypoperfusion. Moreover, alterations in both the central and peripheral immune system are observed, such as microglia activation, the presence of T cells in the brain and the activation of different immune mediators. Hence, neuroinflammation is thought to play a key role in neurodegeneration and cognitive decline [81, 183–186].

The most common animal model to study AD are various transgenic mouse models that overexpress genes involved with familial AD. Some of them mimic the amyloid plaque pathology while others the neurofibrillary tangle pathology, thereby limiting the aspects of this disease that can be studied in an animal model [187]. To date, the effect of the immune system on AD pathogenesis, and specifically that of CD56^bright^ NK cells, remains largely unknown. In this section, we will review the latest literature regarding NK cells in AD and its animal models.

#### Location and migration

Recent research has demonstrated the presence of NK cells in the brains of AD patients, especially in the leptomeninges and CSF, and a few studies have described their presence in AD mouse models [105, 185, 188, 189]. In blood, the frequency of total NK cells as well as that of CD56^bright^ NK cells was similar between AD patients and age-matched healthy controls [190, 191]. Contrarily, a recent paper shows an overall decrease in NK cells in the blood of AD patients [7]. However, the sample size of this study was very small and their healthy controls had a relatively high percentage of NK cells. Future studies with more samples are needed to quantify the frequency of NK cells in AD.

#### Function

Although the studies in humans are limited, some of them suggest a cytotoxic role of circulating NK cells in AD, while others show decreased cytotoxicity [7]. Hence, there are controversial results regarding the function of NK cells in AD pathology.

On one hand, NK cells from AD patients secrete higher levels of TNF and IFN-γ compared to controls, and the release of these cytokines in NK cells correlates negatively with the cognitive function of the patients [6]. Since CD56^bright^ NK cells are known for their cytokine secretion, it could be hypothesized that this effect was mainly due to CD56^bright^ NK cells, but this study did not distinguish between CD56^bright^ and CD56^dim^ NK cells. Furthermore, it has been suggested that the deposits of Aβ in the brain may generate a feedback loop contributing to the secretion of these pro-inflammatory cytokines by NK cells [6]. Aβ can also be recognized by NK cells expressing TLR2 receptors, possibly resulting in an immune response against Aβ, but AD patients with mild symptoms had lower TLR2 expression compared to controls (Fig. 5**)** [190, 192].

On the other hand, no changes were observed in the NK cells when comparing expression of degranulation marker CD107a, inhibitory receptor NKG2A, and activating receptor NKG2D in AD patients with mild symptoms compared to healthy controls (Fig. 5) [190]. Interestingly, in vitro migration of NK cells derived from AD patients towards CCL19 was decreased compared to controls, indicating an impaired homing of the cells to the secondary lymphoid organs [190]. It remains to be established if this is also the case in patients with severe AD and if the migration towards the CNS is also impaired, but it does suggest that NK cells are involved in AD progression, likely with a pathogenic role for the disease.

The triple transgenic mouse model (3xTgAD) is one of the animal models that mimics AD and has peripheral immune cell impairments before the onset of the disease. In this animal model and in contrast to the human studies, the percentage of NK cells in the total leukocyte population is lower already at the age of 2 months, and this percentage is decreased even further at 12 months, indicating that alterations take place in the NK cell population already before the onset of AD [193]. Although depletion of NK cells via anti-NK1.1 antibodies did not affect Aβ levels in this mouse model, it enhanced neurogenesis, reduced neuroinflammation and significantly improved the cognitive abilities of these animals [189]. In another animal model of AD, Rag-5xfAD, which lacks T, B and NK cells, Aβ pathology was twofold increased, microglial density was also increased and their morphology became more amoeboid [194]. Furthermore, microglia had enhanced cytokine production and reduced their phagocytic capacity [194]. However, when this animal model only lacked T and B cells, this microglial phenotype shift was not observed, suggesting an interaction between NK cells and microglia [195]. Together, these results indicate that NK cells might attribute to AD pathogenesis by an increased cytokine secretion, impaired Aβ clearance, dysfunctional migratory capacities and interaction with microglia.

#### Therapies

Very limited literature is available on the effect of different treatments for AD on NK cells, especially due to a lack of effective treatments. The first drug approved by the FDA for AD was Tacrine, which restored the cholinergic function by binding to acetylcholinesterase inhibitors, although it has been discontinued due to side effects [196, 197]. Interestingly, it was the first therapy showing a possible role of NK cells in the pathogenesis of AD, since in vitro treatment of healthy donor-derived NK cells with Tacrine resulted in a suppressed NK cell cytotoxicity [198].

A role for NK cells in AD pathology is further supported by studies on another compound, the anti-inflammatory statin atorvastatin, which is administered to lower cholesterol levels in blood [199]. In the brain of aging rats and rats in which soluble Aβ was injected intracerebroventricularly to mimic AD, expression of CD161 and Nkp46, as well as the level of IFN-γ, was increased, suggesting an increase of NK cells. Upon administration of atorvastatin, both the expression of these NK markers as well as the IFN-γ levels normalized, indicating that atorvastatin reduces NK cells in the brain and might prevent microglial activation by limiting IFN-γ secretion [188]. Furthermore, in these Aβ-injected rats, atorvastatin has also been shown to attenuate neural damage and improve cognitive function [200]. Although functional studies on atorvastatin in human AD patients are lacking, the persistent usage of statins associates with a lower risk for AD [201]. However, more studies on atorvastatin in AD patients are required to establish whether this effect is mediated by the changes in NK cells.

A therapy currently in Phase 1 trial for AD, and Phases 1/2 trial for the treatment of tumors, is SNK01 [202–204]. SNK01 is an autologous cell therapy where NK cells are taken from the patient, expanded and activated in vitro, and later infused back into the patient. Future data from this therapy will help elucidate the role of NK cells in AD.

Together, all these results indicate a pathogenic role for NK cells in AD. However, further research is needed to understand the underlying mechanisms, and potential differences between the effect of CD56^bright^ and CD56^dim^ NK cells and their individual contribution to AD pathogenesis.

### Parkinson’s disease

The main hallmarks of PD are the loss of dopaminergic neurons in the substantia nigra and the accumulation of intracellular aggregates of α-synuclein (α-syn) called Lewy bodies. Extracellular α-syn aggregates are also present in blood and CSF of PD patients, indicating a possible role of α-syn in modulating immune responses in the periphery or the CNS [205]. Patients with PD are characterized by bradykinesia and other diminished motor functions, but also cognitive dysfunction and depression [206, 207]. Two types of PD can be distinguished: early onset and late-onset. Early onset PD patients develop motor symptoms before the age of 40–50, whereas late-onset PD patients show symptoms after the age of 60, and experience a more malignant progression of the disease [208]. Both the adaptive and the innate immune systems play pivotal roles in enhancing neuroinflammation in PD [209, 210]. The disease has a monogenic origin in 10% of all patients, but in the vast majority of patients, PD is caused by a combination of environmental and genetic factors [211]. Accordingly, there are two main approaches to developing animal models to study PD: (1) via neurotoxins and (2) via genetics. In the first approach, different neurotoxins, such as 6-hydroxydopamine (6-OHDA) or 1-methyl-4-phenyl-1,2,3,6-tetrahydropyridine (MPTP) are injected locally or systemically, inducing dopaminergic neuronal loss. In the second approach, transgenic mice carrying mutations for the genes of α-syn (*SNCA*), Parkin (*PRKN*) or Dardarin (*LRRK2*), among others, are used to replicate monogenic or familiar PD [211]. Similarly to AD, it has been suggested that CD56^bright^ NK cells could play both a cytotoxic and a protective role in PD [212]. As the role of NK cells in PD has been recently discussed elsewhere [212, 213], in this review, we will focus on the role of CD56^bright^ NK cells where possible.

#### Location and migration

PD patients have an increased percentage of NK cells out of total lymphocytes in peripheral blood [214, 215]. Contrary to AD research, there is literature available showing NK cells present in the brains of both human PD patients and animal models of PD, especially in close proximity to α-syn aggregates [216]. More recently, an increase in specifically CD56^bright^ NK cells in the substantia nigra has also been shown [217]. NK cells derived from PD patients had decreased levels of VLA-4 expression, suggesting a possible VLA-4-independent migration to the inflamed tissue [218].

#### Function

Until recently, the majority of studies suggested a protective effect of NK cells in PD patients (reviewed elsewhere [213, 219]); however, these studies did not differentiate between CD56^bright^ and CD56^dim^ NK cells. NK cells can internalize and degrade α-syn aggregates, and in the presence of these aggregates, cytotoxicity and IFN-γ secretion of NK cells is reduced (Fig. 5) [216]. As the CD56^bright^ NK cells are the more potent cytokine producers, it could be hypothesized that the reduction of IFN-γ can mostly be attributed to changes in these cells, suggesting a neuroprotective role in PD. However, in a study on microarray expression data of human substantia nigra samples, specifically CD56^bright^ NK cells had a strong correlation with genes involved in the pathogenesis of PD [217]. These preliminary results suggest that CD56^bright^ NK cells are involved in PD pathology, although it remains unclear in which manner.

In addition, depletion of NK cells via the NK1.1. antibody in a PD animal model resulted in an increase of α-syn aggregates in different brain areas and increased pathology, further supporting a beneficial role of NK cells in PD [216]. At the same time, the depletion of NK cells also diminished serum levels of IFN-γ, indicating that NK cells could be the main producers of this cytokine in PD [216]. However, the effect of IFN-γ on PD pathology remains to be established, as well as the contribution to IFN-γ production by CD56^bright^ NK cells. Altogether, these studied indicate a beneficial effect of total NK cells on PD pathology.

Therefore, further research is required to establish the potential differences between the underlying mechanisms of CD56^bright^ and CD56^dim^ NK cells in PD pathology.

#### Therapies

To date, no cure is available for PD, although different treatments exist that are aimed to restore dopamine levels and reduce the motor symptoms of this disease. However, in the long run, these treatments lose efficacy and may cause serious side effects [220]. The most common drug used for the treatment of PD is levodopa (L-DOPA), a precursor of dopamine [220]. However, levodopa’s bioavailability in the brain is scarce and different methods are currently being developed to facilitate the crossing of the BBB and improve treatment efficacy [218, 221]. The percentage of NK cells in PD patients does not correlate with daily dose of levodopa, suggesting that levodopa does not affect the percentage nor counts of CD56^+^ cells [214, 222]. However, levodopa increases serum IL-15 in PD patients compared to healthy controls [223], thereby CD56^bright^ NK cells could become more active after levodopa treatment. Yet, dopamine inhibits the effector function of activated NK cells, such as IFN-γ secretion [224]. Thus, it could be possible that levodopa treatment in PD also inhibits activated NK cells.

Interestingly, a new clinical trial will be conducted to study the effect of SNK01, the autologous cell therapy where activated NK cells are infused back to the patient, for the treatment of PD, as similarly being done in AD [202, 225]. Expanding NK cells could enhance the degradation of α-syn aggregates in the brain and thereby reduce disease pathogenicity. However, there is currently no indication on whether this new therapy leads to an expansion of NK cells in general or preferentially that of CD56^bright^ or CD56^dim^ NK cells.

### Amyotrophic lateral sclerosis

ALS is a neurodegenerative disorder characterized by the loss of motor neurons and consequently, it affects the control of voluntary muscles. ALS is a relentlessly progressive disease, causing mortality in the majority of cases within 2–5 years from the onset of the symptoms [226]. Familial or genetic ALS is caused by mutations in different genes such as *C9orf72*, TAR DNA binding protein (*TARDBP*), superoxide dismutase (*SOD1*) and FUS RNA binding protein (*FUS*), and accounts for 10–15% of the cases [227]. However, the majority of the patients suffer from sporadic ALS, for which the cause remains unknown. The molecular pathophysiology is not well understood but has been classified into four major categories: altered RNA metabolism, cytoskeletal and trafficking defects, impaired proteostasis or autophagy and mitochondrial dysfunction [226]. The immune system is an important contributor to this disease, but it has been suggested that the immune system has a double-edged involvement characterized by an early anti-inflammatory and protective response, followed by a pro-inflammatory and cytotoxic late response [228].

The majority of animal models used to study ALS are based on known mutations in the aforementioned genes, and hence, represent familial ALS. For sporadic ALS, there is a lack of animal models that mimic the disease, although ALS animal models based on environmental factors are being developed. For example, the exposure of mice to phytosterol glucosides, such as β-Sitosterol-β-d-glucoside, leads to progressive motor neuron degeneration and represents many aspects of the pathology in humans, although there are still limitations and caveats since in rats, pathology upon exposure to this compound closely resembles more PD than ALS [229].

#### Location and migration

Regarding the percentage of peripheral NK cells in ALS compared to controls, results are conflicting, with some studies showing an increase and others a decrease [230, 231]. However, the proportion of peripheral CD56^bright^ NK cells is increased in ALS patients compared to controls [230]. Accordingly, there is also an increase of serum IFN-γ in those patients that could be produced by CD56^bright^ NK cells [230].

Peripheral NK cells from ALS patients had increased expression of trafficking markers, such as CD11a (subunit of LFA-1), CD11b (or integrin α-M) and CX3CR1, suggesting a possible migration towards the spinal cord and motor cortex in ALS donors (Fig. 5) [231, 232]. Indeed, in postmortem spinal cord and cerebral motor cortex tissue, NKp46^+^ cells were detected in sporadic ALS patients but not in controls [231]. Intriguingly, ALS patients with slower disease progression presented increased numbers of CD56^bright^ NK cells in the CSF compared to patients with rapid disease progression [233]. Together, these results suggest that NK cells, and possibly CD56^bright^ NK cells specifically, migrate into the CNS in ALS and exert a neuroprotective function.

Similar results were obtained in an ALS mouse model with a mutation in SOD1 (hSOD1^G93A^), in which an increase of the percentage of NK cells in total lymphocytes was observed in the spinal cord and motor cortex early in disease, whereas these levels declined upon further disease progression [231]. Depleting the chemokine CCL2, known to be increased in ALS patients and involved in CD56^bright^ NK cell recruitment, significantly reduced the percentage of NK cells in the CNS of these mice during early disease, suggesting a role for this chemokine in the migration of NK cells into the CNS in ALS [231].

#### Function

In humans, the functions of NK cells during ALS, and specifically CD56^bright^ NK cells, are still unknown. However, there are a few indications and hypotheses regarding their role. The ratio of CD4^+^, CD8^+^ T cells and monocytes divided by the amount of CD56^bright^ NK cells could be used to classify ALS patients with fast or slow progression [233]. Furthermore, higher expression levels of CD56^+^CD16^−^ on peripheral NK cells are associated with a lower risk of ALS [234]. As mentioned previously, patients with a more rapid disease progression had lower levels of CD56^bright^ NK cells in the CSF compared to patients with a slower disease progression, suggesting a protective role of CD56^bright^ NK cells in ALS [233]. As activated T cells in the periphery and CSF are thought to exacerbate ALS pathogenesis, the potentially protective effect of CD56^bright^ NK cells could possibly be attributed to the regulation of T cells by CD56^bright^ NK cells, similarly as in MS. In ALS patients with rapid disease progression, CD56^bright^ NK cells might not be able to limit the responses of T cells, resulting in increased disease progression [233].

Depleting NK cells in ALS animal model has resulted in conflicting data, probably due to the timing of the depletion and the sex of the animals. In the ALS animal model hSOD1^G93A^, depletion of NK cells via NK1.1 antibody at 8,5 weeks or 13 weeks resulted in a decrease of CNS NK cell levels but did not affect survival rates or peripheral immune cell counts [232, 235]. Interestingly, stratification by sex showed that NK cell depletion increased the survival of female ALS mice, but no effects were seen in male mice [232]. This demonstrates that in ALS, sex-specific immune differences are likely present. Nevertheless, another study has shown that depletion of NK cells at 8 weeks in the same animal model hSOD1^G93A^ and another animal model (TDP43^A315T^) delayed the onset of motor impairment and increased the survival time, but there was no effect on disease progression [231]. In addition, neurons from the hSOD1^G93A^ mice overexpressed *mult-1* and *nectin-2* in comparison with wild-type mice. *Mult-1* and *nectin-2* are ligands for NKG2D and DNAM-1, respectively, which trigger the cytotoxicity of NK cells. This increased cytotoxicity of NK cells in response to these neurons of hSOD1^G93A^ mice was confirmed by co-culturing these neurons with NK cells isolated from the spleen of wild type mice, which resulted in an increased percentage of degranulating CD107a^+^ NK cells and decreased viability of hSOD1^G93A^ neurons [231].

To summarize these contradictory results, in the early stages of ALS, NK cells seem to have a neurotoxic role in the ALS animal models while a neuroprotective role is indicated for CD56^bright^ NK cells in humans. Hence, and as suggested for the other neurodegenerative diseases mentioned in this review, future research will need to characterize NK cell subsets both in humans and animal models to clarify these clashing results and unravel the role of NK cells in ALS pathogenesis.

#### Therapies

Due to all the conflicting results, it is difficult to speculate if an upregulation of CD56^bright^ NK cells in ALS patients may be successful in limiting disease progression. To date, only three drugs that target different symptoms of the disease and provide a modest clinical benefit have been approved by the FDA for the treatment of ALS: riluzole, edaravone and just recently, AMX0035 [236–238]. Riluzole is a glutamate release inhibitor that blocks voltage-dependent sodium channels, which seems to have greater benefits at an early stage of ALS, before the loss of motor neurons [239]. To our knowledge, no research has been done to study the effect of riluzole on NK cells, mainly because the majority of studies mentioned in the previous section included patients that were allowed to take riluzole.

Tofacitinib is a small molecule used for the treatment of different autoimmune diseases that recently has also been studied in the context of ALS [240]. This drug blocks the signaling pathway of IL-15, one of the cytokines involved in NK cell survival and function, and thereby reduces NK cell numbers in blood [241]. Tofacitinib suppressed the cytotoxicity and secretion of pro-inflammatory cytokines by ALS patients-derived NK cells. Furthermore, and more importantly, treating the NK cell-line NK-92 with tofacitinib resulted in decreased cytotoxicity of these NK cells against iPSC-derived neurons derived from an ALS patient, as neuronal cell death was significantly decreased [240]. Therefore, this drug is potentially a viable therapy for ALS by suppressing NK cell function and reducing motor neuron loss. However, these studies did not differentiate between CD56^bright^ NK cells and CD56^dim^ NK cells, and this could have great repercussions for the treatment of ALS since the neuroprotective role of CD56^bright^ NK cells and especially, the timing of their neuroprotection is still under debate.

### Future perspectives

In this review, we provided an updated perspective on the role of CD56^bright^ NK cells in neurodegenerative diseases and discussed the limitations of animal models and studies overlooking NK cell subset differentiation. It is tempting to speculate that CD56^bright^ NK cells, being immunomodulatory cells, have a neuroprotective role in autoimmune diseases and a neurotoxic role in diseases in which a potent immune response is desired. However, in this review we show that the effect of CD56^bright^ NK cells on pathogenesis is often more complex. We also illustrate how animal models can be useful and yet yield contradictory results, partially due to the absence of corresponding markers.

Current immunotherapies might deplete or target the entire NK cell population but depending on the specific pathophysiology in different neurodegenerative diseases, specifically targeting CD56^bright^ or CD56^dim^ NK cells should be taken into consideration.

Hence, future studies are needed to elucidate how the distinct NK cell subsets are involved in the various diseases and throughout disease progression. Next research should first determine the distinct expression of markers on CD56^bright^ and CD56^dim^ NK cells in both blood and the CNS to allow for the identification of a selective druggable targets for the distinct subsets. This is especially important since NK subsets might also act as a double-edged sword in certain diseases. For example, in MS, the expansion of CD56^bright^ NK cells might lead to the suppression of autoreactive T cells, but also to a harmful increase in IFN-γ production. Therefore, uncovering and targeting specific underlying pathways will increase treatment efficacy and reduce the risk of potential side effects. Altogether, this also brings up a new challenge: if CD56^bright^ NK cells will eventually mature into CD56^dim^ NK cells, how can we target one subset without affecting the other? Nevertheless, although the role of CD56^bright^ NK cells in different neurodegenerative diseases is still speculative, there is mounting evidence suggesting their importance in disease pathogenesis.

## Data Availability

Not applicable.

## Abbreviations

6-OHDA: 6-Hydroxydopamine
α-syn: α-Synuclein
Aβ: Amyloid-β
AD: Alzheimer’s disease
ADCC: Antibody-dependent cellular cytotoxicity
ALS: Amyotrophic lateral sclerosis
APOE4: Apolipoprotein ε4 allele
APP: Amyloid precursor protein
BBB: Blood brain barrier
BCSFB: Blood–cerebrospinal fluid barrier
BMB: Blood–meningeal barrier
CAM: Cellular adhesion molecule
CCL: Chemokine C–C motif ligand
CCR: C–C chemokine receptor
ChAT: Choline acetyltransferase
CNS: Central nervous system
CSF: Cerebrospinal fluid
CXCR: C–X–C chemokine receptor
DMTs: Disease-modifying therapies
EAE: Experimental autoimmune encephalomyelitis
FUS: FUS RNA binding protein
HBMECs: Human brain microvascular endothelial cells
ICAM-1: Intercellular adhesion molecule 1
IFN: Interferon
IL: Interleukin
ILCs: Innate lymphoid cells
KIRs: Killer-cell immunoglobulin-like receptors
L-DOPA: Levodopa
LTi: Lymphoid tissue inducer cells
LFA-1: Lymphocyte function-associated antigen 1
MBP: Myelin basic protein
MHC: Major histocompatibility complex
MOG: Myelin–oligodendrocyte glycoprotein
MPTP: 1-Methyl-4-phenyl-1,2,3,6-tetrahydropyridine
MS: Multiple sclerosis
NCRs: Natural cytotoxicity receptors
NK: Natural killer cells
NKG: Natural killer group
PD: Parkinson’s disease
PD-1: Programmed cell death protein 1
PD-L1: Programmed death-ligand 1
PLP: Myelin proteolipid protein
PMA: Phorbol myristate acetate
PNS: Peripheral nervous system
PPMS: Primary progressive multiple sclerosis
PS1-2: Presenilin 1 or 2
PSGL-1: P-selectin glycoprotein ligand-1
RAG: Recombination activating genes
ROS: Reactive oxygen species
RRMS: Relapsing–remitting multiple sclerosis
SOD1: Superoxide dismutase
SPMS: Secondary progressive multiple sclerosis
TARDBP: TAR DNA binding protein
Th: T helper cells
TNF: Tumor necrosis factor
TRAIL: TNF-related apoptosis-inducing ligand
VCAM-1: Vascular cell adhesion molecule 1
VLA-4: Very late activation antigen-4
